# Supplementary material for: Spatial mapping and determinants of intimate partner violence among married women in Sierra Leone: A cross-sectional study
Source: PLoS One. 2025 May 28;20(5):e0325360. doi: 10.1371/journal.pone.0325360 (PMC12118840; doi:10.1371/journal.pone.0325360)
Supplement: Attachment — Submitted filename: Review Report for Manuscript PONE.pdf [file pone.0325360.s001.pdf]

Review Report for Manuscript PONE-D-25-03253

Title: Spatial Mapping and Determinants of Intimate Partner Violence among Married Women in Sierra Leone

General Assessment: This study addresses an important public health and human rights issue by analyzing the spatial distribution and determinants of intimate partner violence (IPV) among married women in Sierra Leone. The research utilizes data from the 2019 Sierra Leone Demographic and Health Survey (SLDHS), applying spatial autocorrelation analysis and mixed-effect multilevel binary logistic regression. The findings provide valuable insights into the geographic clustering of IPV and its associated socio-demographic and behavioral factors. However, there are several areas that require clarification, methodological improvement, and a stronger theoretical grounding.

Major Concerns:

1. **Justification for Age Group Selection:**
    - The study includes married women aged 15-49 but emphasizes results for the 25-29 age group. A clearer justification for this focus is needed. Why was this subgroup emphasized over others?
    - The paper should discuss potential biases due to the exclusion of unmarried women who may also experience IPV.
2. **Methodological Clarity:**
    - The mixed-effects multilevel binary logistic regression approach needs more detailed explanation regarding the selection of variables in different models.
    - Provide a justification for why only certain socio-demographic variables were considered, and discuss if any variables were excluded due to collinearity or missing data.
    - The study mentions the use of "Empirical Bayesian Kriging (EBK)" for spatial interpolation. The rationale behind selecting this technique over others should be explicitly discussed.

3. **Ethical Considerations:**
    - The study states that "Ethical clearance was not sought" due to the secondary nature of the data. However, IPV is a sensitive topic, and there should be a discussion of how ethical considerations were managed in handling potentially distressing content.
    - Explain whether the data were anonymized and if informed consent was obtained in the original survey.
4. **Statistical Interpretation and Model Fit:**
    - The reported odds ratios in Table 5 require a more nuanced interpretation. Some variables (e.g., education, wealth index) were found to be non-significant. The discussion should provide possible explanations.
    - Discuss the model's goodness-of-fit (e.g., AIC, BIC, likelihood ratio tests) and justify why the final model was chosen.
5. **Comparative Analysis with Existing Literature:**
    - The introduction and discussion reference global and regional IPV prevalence rates, but there is limited comparison with prior studies in Sierra Leone.
    - The authors should engage more critically with existing research on IPV determinants in Sub-Saharan Africa and discuss how their findings align or differ from past studies.
6. **Implications and Recommendations:**
    - The conclusion suggests policy interventions but remains somewhat generic. More concrete, actionable recommendations are needed.
    - Consider discussing how geographic mapping of IPV could be used for targeted interventions by local authorities and NGOs.
    - Address the feasibility of implementing policy changes in Sierra Leone's socio-political context.

Minor Concerns and Suggestions:

1. **Grammar and Clarity:**
   - There are minor grammatical issues throughout the manuscript. A thorough proofreading is recommended.
   - Example: In the results section, the sentence "The study revealed an overall IPV prevalence of 56%, with physical, sexual, and emotional violence accounting for 38%2%, 6.2%, and 45.9%, respectively." should be corrected for clarity.
2. **Figure and Table Presentation:**
   - Some tables (e.g., Table 3) are dense and difficult to interpret. Consider simplifying them by only including significant variables or restructuring them for better readability. **I suggest replacing Table 3 with a forest plot, as it offers a more intuitive and visually informative presentation of the data. This will enhance clarity and improve the interpretability of the results. (sample forest plot attached)**
   - Figures depicting spatial clusters of IPV are useful but should include a more detailed legend and explanation of statistical significance.
3. **Terminology and Conceptual Consistency:**
   - The manuscript sometimes alternates between "IPV" and "domestic violence"—ensure consistency in terminology throughout.
   - The terms "hotspots" and "cold spots" should be clearly defined in the methodology.

Conclusion:

The manuscript makes a valuable contribution to the literature on IPV by incorporating spatial analysis, but improvements in methodological clarity, statistical interpretation, and policy recommendations are necessary. Addressing the above concerns will strengthen the study's impact and applicability. I encourage the authors to revise the manuscript accordingly and provide detailed responses to the points raised in their rebuttal letter.

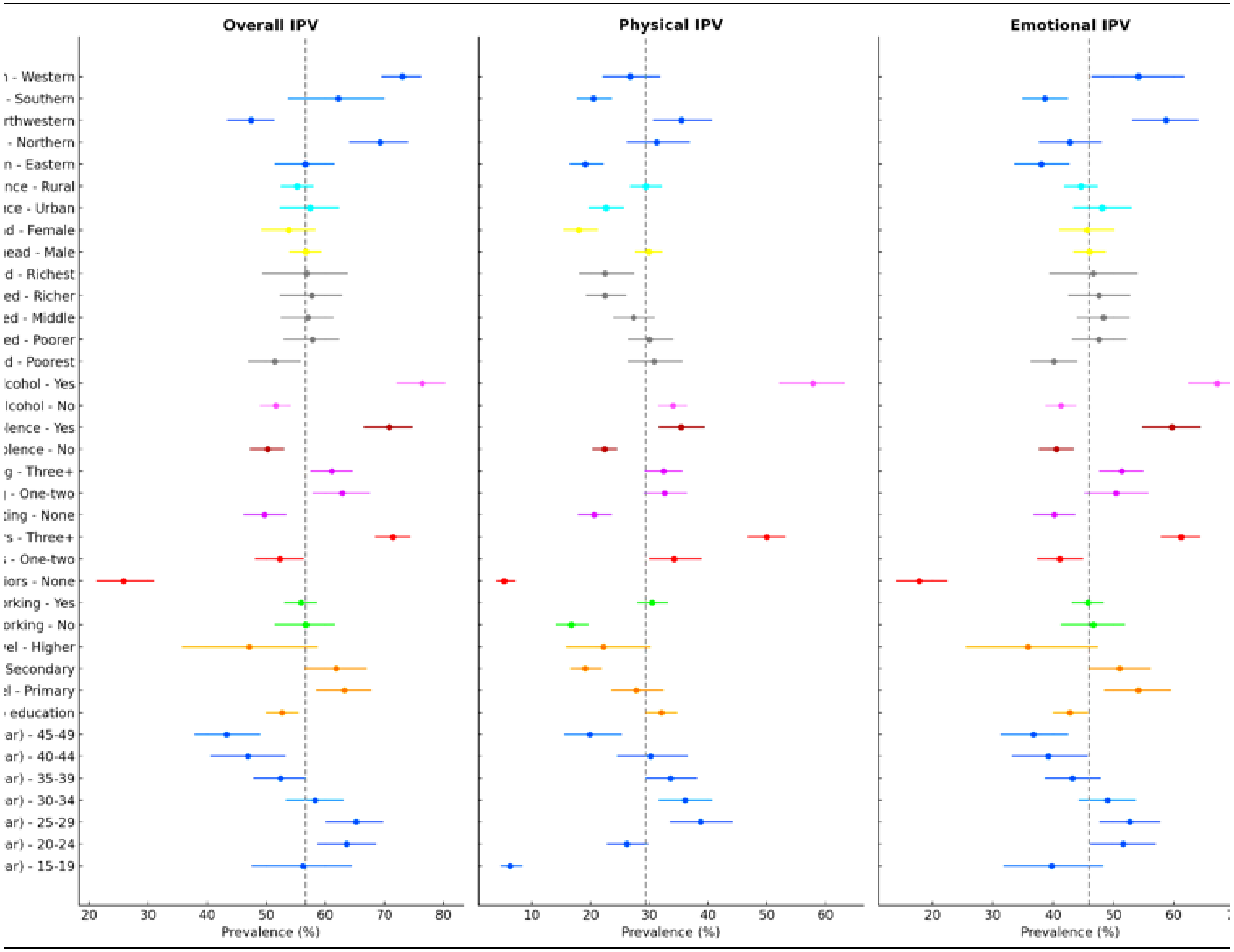

Sample forest plot generated by “Matplotlib” in Python
