# Supplementary material for: Spatial mapping and determinants of intimate partner violence among married women in Sierra Leone: A cross-sectional study
Source: PLoS One. 2025 May 28;20(5):e0325360. doi: 10.1371/journal.pone.0325360 (PMC12118840; doi:10.1371/journal.pone.0325360)
Supplement: Attachment — Submitted filename: Response to Reviewers.docx [file pone.0325360.s003.docx]

The Editor

PLOS ONE

11 th April 2025

Ref: PONE-D-24-25104

Title: Spatial Mapping And Determinants of Intimate Partner Violence Among

Married Women In Sierra Leone.

Response to Reviewers&#39; comments

Dear Sir/Madam,

We want to express our sincere thanks for painstakingly reviewing our manuscript

and providing valuable comments and suggestions. Please see our point-by-point

response to the reviewers&#39; comments and suggestions. Revisions are highlighted

with track changes in the revised manuscript.

Journal Requirements:

When submitting your revision, we need you to address these additional

requirements.

1. Please ensure that your manuscript meets PLOS ONE&#39;s style requirements,

including those for file naming. The PLOS ONE style templates can be found at

https://journals.plos.org/plosone/s/file?id=wjVg/PLOSOne_formatting_sample_main_

body.pdf and

https://journals.plos.org/plosone/s/file?id=ba62/PLOSOne_formatting_sample_title_a

uthors_affiliations.pdf

**Response: Thank you. We have ensured that our manuscript meets PLOS ONE style requirements.**

2. We note that Figure 1 and 2 in your submission contain [map/satellite] images

which may be copyrighted. All PLOS content is published under the Creative

Commons Attribution License (CC BY 4.0), which means that the manuscript,

images, and Supporting Information files will be freely available online, and any third

party is permitted to access, download, copy, distribute, and use these materials in

any way, even commercially, with proper attribution. For these reasons, we cannot

publish previously copyrighted maps or satellite images created using proprietary

data, such as Google software (Google Maps, Street View, and Earth). For more

information, see our copyright guidelines: http://journals.plos.org/plosone/s/licenses-

and-copyright.

We require you to either (1) present written permission from the copyright holder to

publish these figures specifically under the CC BY 4.0 license, or (2) remove the

figures from your submission:

a. You may seek permission from the original copyright holder of Figure 1 and 2 to

publish the content specifically under the CC BY 4.0 license.

We recommend that you contact the original copyright holder with the Content

Permission Form (http://journals.plos.org/plosone/s/file?id=7c09/content-permission-

form.pdf) and the following text:

“I request permission for the open-access journal PLOS ONE to publish XXX under

the Creative Commons Attribution License (CCAL) CC BY 4.0

(http://creativecommons.org/licenses/by/4.0/). Please be aware that this license

allows unrestricted use and distribution, even commercially, by third parties. Please

reply and provide explicit written permission to publish XXX under a CC BY license

and complete the attached form.”

Please upload the completed Content Permission Form or other proof of granted

permissions as an &quot;&quot;Other&quot;&quot; file with your submission.

In the figure caption of the copyrighted figure, please include the following text:

“Reprinted from [ref] under a CC BY license, with permission from [name of

publisher], original copyright [original copyright year].”

b. If you are unable to obtain permission from the original copyright holder to publish

these figures under the CC BY 4.0 license or if the copyright holder’s requirements

are incompatible with the CC BY 4.0 license, please either i) remove the figure or ii)

supply a replacement figure that complies with the CC BY 4.0 license. Please check

copyright information on all replacement figures and update the figure caption with

source information. If applicable, please specify in the figure caption text when a

figure is similar but not identical to the original image and is therefore for illustrative

purposes only.

The following resources for replacing copyrighted map figures may be helpful:

USGS National Map Viewer (public domain): http://viewer.nationalmap.gov/viewer/

The Gateway to Astronaut Photography of Earth (public

domain): http://eol.jsc.nasa.gov/sseop/clickmap/

Maps at the CIA (public domain): https://www.cia.gov/library/publications/the-world-

factbook/index.html and https://www.cia.gov/library/publications/cia-maps-

publications/index.html

NASA Earth Observatory (public domain): http://earthobservatory.nasa.gov/

Landsat: http://landsat.visibleearth.nasa.gov/

USGS EROS (Earth Resources Observatory and Science (EROS) Center) (public

domain): http://eros.usgs.gov/#

Natural Earth (public domain): http://www.naturalearthdata.com/

**Response:** Thank you for your input regarding Figures 1 and 2 in our manuscript. We confirm that both figures were entirely generated by us using publicly available datasets from the Spatial Data Repository, The Demographic and Health Surveys Program (ICF International), funded by the United States Agency for International Development (USAID). The data was accessed from [spatialdata.dhsprogram.com](https://spatialdata.dhsprogram.com) on December 4, 2024, as cited in the figure captions.

Since we created these figures, they are original works and do not infringe on any third-party copyright. We hereby confirm that we are the copyright holder of these figures and grant permission for their publication under the Creative Commons Attribution License (CC BY 4.0). We have ensured that the figure captions include the appropriate attribution and licensing information.

"Source: Spatial Data Repository, The Demographic and Health Surveys Program, ICF International. Funded by the United States Agency for International Development (USAID). Available from spatialdata.dhsprogram.com. [Accessed 04 December 2024]. Created by the author and published under the CC BY 4.0 license."

**Reviewer #1:** ### 1. Measuring Outcome Variables

How do you measure your outcome variable? Have you employed the same

measurement for both spatial and multilevel analyses?

**Response:**

**We appreciate the reviewer's insightful question concerning the measurement of our outcome variable and its application in both spatial and multilevel analyses. Our study measure intimate partner violence (IPV) among married women utilizing the standardized Domestic Violence Module from the 2019 Sierra Leone Demographic and Health Survey (DHS).**

**This module collects self-reported data on various forms of IPV, including physical, emotional, and sexual violence, experienced by women aged 15–49 who are currently or previously married or in a union. The IPV variable is typically constructed by aggregating responses to specific questions regarding acts such as being slapped, pushed, hit, kicked, forced into sexual acts, or insulted by a partner.** **The responses are coded into a binary composite measure (0 = no IPV, 1 = any IPV), in line with established DHS methodology and prior studies (Seidu, Mossie). This coding indicates whether a woman has experienced any form of intimate partner violence (IPV) within a specified timeframe, such as the past 12 months or at any point in her life. This approach allows researchers to quantify and analyze the prevalence and patterns of IPV among married women in Sierra Leone.**

**We employed a consistent measure of intimate partner violence (IPV) for both spatial and multilevel analyses to ensure the comparability of our findings. In the spatial analysis, we linked each of the three types of IPV—Physical, Emotional, and Sexual violence—represented as binary variables, to the GPS coordinates of DHS clusters. This approach allowed us to identify geographic patterns while accounting for spatial autocorrelation. For the multilevel analysis, we maintained the same binary outcome (IPV) but focused on differentiating between individual and community-level factors using hierarchical random effects.**

\### 2. Choice of Spatial Interpolation Techniques

There are numerous spatial interpolation techniques, such as Inverse Distance Weighting (IDW), Kriging, splines, and natural neighbors. Why did you choose

Bayesian spatial Kriging? What are the implications and added value of using this

model in the context of your findings?

**Response:**

**We extend our sincere gratitude to the reviewer for their insightful inquiry regarding our selection of Bayesian spatial Kriging as the methodological framework for this study. Our choice to employ this particular spatial interpolation technique is underpinned by several theoretical and practical considerations that render it particularly suitable for investigating the dynamics of intimate partner violence (IPV) in Sierra Leone.**

The Bayesian approach to spatial Kriging offers significant advantages over traditional interpolation methods, such as Inverse Distance Weighting (IDW) or frequentist Kriging, particularly in the context of complex health outcome data. Notably, empirical Bayesian Kriging (EBK) excels in mitigating variability and predicting values in regions that remain unsampled by exploiting data points from neighboring locales. This technique harnesses the principles of spatial autocorrelation to discern spatial trends within the data and to forecast the underlying dependency structures[38]. The method incorporates simulations and subsetting paradigms for parameter estimation, deriving the semivariogram from existing data points, thereby enabling the estimation of values at unobserved locations using a unified semivariogram. It is crucial to acknowledge that this approach presumes the semivariogram calculated for the interpolation zone accurately represents the true semivariogram [39].

In comparative assessments against alternative spatial interpolation techniques, Bayesian Kriging has demonstrated superior performance tailored to our specific research imperatives. EBK has exhibited enhanced accuracy through the integration of auxiliary variables together with spatial autocorrelation[40]. Furthermore, Bayesian Kriging addresses the tendency of Ordinary Kriging to produce overly optimistic prediction variances by conceptualizing model parameters as random variables, which facilitates more robust uncertainty quantification[41].

The implementation of Bayesian spatial Kriging has profoundly influenced our findings, establishing it as an invaluable tool for the exploration of patterns and risk factors associated with IPV. Numerous studies have successfully employed this methodology to identify high-risk areas in conjunction with pertinent neighborhood-level characteristics[42-45]. Moreover, this modeling approach adeptly identifies hotspots, regions characterized by uncertain estimates, and covariates that elucidate the spatial patterns of the outcome variable[43]. The Bayesian spatial modeling framework has enabled researchers to account for both fixed covariate effects and spatially structured random effects, thereby facilitating a comprehensive understanding of IPV patterns and informing targeted prevention strategies[44, 45]. We recognize that the application of Bayesian spatial techniques presents computational challenges and necessitates meticulous selection of prior distributions. To address these concerns, we undertook extensive sensitivity analyses to evaluate the influence of varying prior specifications on our study's findings.

\### 3. Geographic Boundaries and Units of Analysis

What geographic boundaries or unit levels were used in each analysis? Based on

the DHS data, these could be at the level of countries, regions (comprising multiple

countries) within Sub-Saharan Africa (SSA), census clusters, or GPS points of

households. It is crucial to clarify this for accurate interpretation of the spatial

analysis.

**Response:**

**We greatly appreciate the reviewer's question regarding the geographic units used in our analyses. Clarifying these boundaries is indeed crucial for accurate interpretation of both our spatial and multilevel findings. In our study, we used sub-national regional boundaries for the SLDHS, specifically we used administrative level 2 that comprises of 16 districts to maximize analytical precision while maintaining relevance for policy implementation in Sierra Leone.**

Your findings indicated a district-level analysis. How did you define districts?

Specifically, how did you identify the number of districts, and you mentioned 18

districts—how did you determine this? How many districts were included in the

study?

**Response:**

**We thank the reviewer for this question regarding our district-level analysis. We appreciate the opportunity to clarify how we defined and incorporated districts into our study. In our analysis, we used Sierra Leone’s administrative boundaries at Level 2 (districts)** as defined in the **2015 Population and Housing Census (Statistics Sierra Leone, 2016) and aligned with the 2019 Sierra Leone Demographic and Health Survey (SLDHS) geographic coding. Additionally, we included the 16 districts, which served as the primary sub-national administrative units for policy implementation and health planning.**

You utilized DHS data, which is derived from a spatial repository that includes

enumeration areas for specific countries. How did this data translate into district-level

analysis? Please provide a clear description of the level of analysis used.

**Response:**

**We appreciate the reviewer's question regarding how we translated the DHS spatial data into district-level analysis. This is a crucial methodological consideration, and we added a detailed explanation of our approach in the main manuscript from lines 109 to 124 of page 7.**

\### 4. Managing Missing Data

How do you manage missing data, including missing attributes and missing

coordinate data?

**Response:**

**We thank you for your comment. We addressed missing data through multiple imputations for non-essential missing values and implemented complete case analysis when the amount of missing data was minimal. Furthermore, we utilized DHS sample weights to adjust for non-response, ensuring that our results were representative. Finally, we acknowledge the DHS program's geographic displacement protocol which occasionally results in missing or unreliable coordinates for sensitive locations, we therefore appended the IPV-related dataset with the Global Positioning System (GPS) coordinates of SLDHS and then conducted sensitivity analyses comparing results with and without append GIS.**

\### 5. Study Variables

Please elaborate on your outcome variable. Have you categorized it into binary

responses (yes or no)? Clearly indicate this.

**Response:**

**We appreciate the reviewer's question regarding the outcome varaible. We made a clear explanation of the outcome varaible in the main manuscript from lines 127 to 140 of pages 7 and 8.**

\### 6. Spatial Autocorrelation

Which spatial matrix did you use to report spatial autocorrelation (e.g., Moran&#39;s I)?

Please articulate the implications based on your findings.

**Response:**

**We thank the reviewers for the spatail amtrix use to report the spatil autocorrelation. In our study, to assess the spatial pattern of IPV across Sierra Leone we** conducted a spatial autocorrelation analysis using **Global Moran's I** with an **inverse-distance spatial weights matrix**. This approach allowed us to evaluate whether IPV prevalence exhibited clustering, dispersion, or random distribution across districts. The inverse-distance matrix was selected because it assumes that nearby districts exert greater influence on each other than those farther apart—a theoretically plausible assumption for social phenomena like IPV, where cultural norms and resource availability often follow geographic gradients.

\### 7. Multi-Level Analysis

You conducted Intraclass Correlation Coefficient (ICC) analysis. Given that your

analysis is multi-level, it would be beneficial to include Measures of Relative Risk

(MOR) and Proportional Change in Variance (PCV). What were your community-

level and individual-level variables?

**Response:** This study included fourteen independent variables selected based on their availability in the dataset and insights from prior research. Individual-level variables comprised the respondent's age, educational level, employment status, exposure to family planning through television, radio, and newspapers, partner's controlling behaviors, justification of spousal abuse, interparental violence, and husband's alcohol consumption. Community-level variables included gender of the household head, wealth index, residential location (urban/rural) and geographical region.

**Thank you for your suggestion regarding the inclusion of Measures of Relative Risk (MOR) and Proportional Change in Variance (PCV) in our analysis. We appreciate the importance of these measures in multi-level modeling and understand their utility in interpreting community-level effects. However, we believe the current analysis, which includes the Intraclass Correlation Coefficient (ICC) and random effect modeling, is sufficient to address the objectives of this study.**

**The ICC provides a robust measure of the proportion of variance attributable to community-level factors, which is critical in understanding the clustering of intimate partner violence (IPV) within communities. By reporting PSU variance alongside ICC values, we have effectively demonstrated the extent of variability at the community level. Furthermore, the Wald chi-square test, log-likelihood values, and AIC scores collectively provide sufficient information on model fitness and the significance of explanatory variables.**

**While MOR and PCV are useful for quantifying relative risk and proportional changes in variance, these measures are primarily recommended when the focus is on interpreting the strength of community-level effects relative to individual-level factors. In our study, the primary aim was to identify significant determinants of IPV at both levels and assess overall model performance, which the current analysis adequately achieves. Including MOR and PCV would add complexity without substantially enhancing the interpretability of our findings.**

You mentioned explanatory variables; please elaborate further. When performing a

multi-level analysis, it is essential to differentiate between these variables.

Response:

Thank you for your suggestions. We have eleborated further by differentiating the explanatory varibles in the main manuscript from lines 143 to 146 of page 8.

Reviewer #2: I appreciate the opportunity to review this significant study, which

explores an important public health and human rights issue by analyzing the spatial

distribution and determinants of intimate partner violence (IPV) among married

women in Sierra Leone. Please find my detailed comments attached.

Response:

We sincerely appreciate the reviewer’s engagement with our study and the recognition of its significance in addressing IPV as both a public health and human rights concern in Sierra Leone. We are grateful for the time taken to provide detailed comments, which will help strengthen our manuscript.

**Review Report for Manuscript PONE-D-25-03253**

**Title:** Spatial Mapping and Determinants of Intimate Partner Violence among Married

Women in Sierra Leone

**General Assessment**: This study addresses an important public health and human

rights issue by analyzing the spatial distribution and determinants of intimate partner

violence (IPV) among married women in Sierra Leone. The research utilizes data

from the 2019 Sierra Leone Demographic and Health Survey (SLDHS), applying

spatial autocorrelation analysis and mixed-effect multilevel binary logistic regression.

The findings provide valuable insights into the geographic clustering of IPV and its

associated socio-demographic and behavioral factors. However, there are several

areas that require clarification, methodological improvement, and a stronger

theoretical grounding.

**Response:**

**We thank the reviewer the positive feeback and the valuable suggestion for strengthening our study.**

**Major Concerns:**

1. Justification for Age Group Selection:

o The study includes married women aged 15-49 but emphasizes results

for the 25-29 age group. A clearer justification for this focus is needed.

Why was this subgroup emphasized over others?

**Response:** **The study includes married women aged 15–49 years to examine the prevalence and determinants of Intimate Partner Violence (IPV) across the reproductive age range. While all age groups were analyzed, the 25–29 age group was emphasized because it was the only group significantly associated with IPV in the adjusted models, with an aOR of 1.90 [95% CI: 1.15–3.16] in Model II and 1.76 [95% CI: 1.06–2.93] in Model IV. Other age groups, such as 30–34 years and older, did not show statistically significant associations, as their confidence intervals included 1. Highlighting the 25–29 age group ensures clarity and reflects the most meaningful results of the study, as this finding may indicate unique vulnerabilities or risk factors for IPV within this subgroup, warranting further exploration and targeted interventions.**

o The paper should discuss potential biases due to the exclusion of

unmarried women who may also experience IPV.

**Response:** **The study focuses exclusively on married women aged 15–49 years, which may introduce potential biases by excluding unmarried women who may also experience Intimate Partner Violence (IPV). Unmarried women, including those in cohabiting relationships or dating partnerships, are also at risk of IPV, and their exclusion may limit the generalizability of the findings to all women of reproductive age. This exclusion could result in an underestimation of the overall prevalence of IPV, as unmarried women may face different dynamics of abuse, including those related to less formalized or shorter-term relationships. Additionally, cultural and societal factors influencing IPV may differ between married and unmarried women, potentially leading to variations in risk factors and experiences of IPV. Future studies should aim to include unmarried women to provide a more comprehensive understanding of IPV across diverse relationship contexts.**

**2. Methodological Clarity:**

o The mixed-effects multilevel binary logistic regression approach needs

more detailed explanation regarding the selection of variables in

different models.

**Response:**

We appreciate the reviewer’s the reviewer's request for additional clarification regarding our model-building strategy. Our sequential approach to the mixed-effects multilevel binary logistic regression analysis was carefully designed to progressively examine how different levels of factors contribute to IPV risk while maintaining methodological rigor.

We evaluated the distribution of the explanatory variables concerning the result variable and utilised a Pearson chi-square test to illustrate their relationships. However, we selected only independent varaibles that were statistically significant with a p-value of less than 0.05 from the IPV bivariate analysis for the mixed-effects multilevel binary logistic regression analysis . A multicollinearity evaluation was performed among the independent variables using the variance inflation factor (VIF) before the regression analysis. The findings demonstrated that none of the variables analysed had considerable collinearity, as the value was below five.

Model I, devoid of explanatory factors, illustrated the changes in IPV among married women due to clustering at the primary sampling units (PSU), allowing us to quantify the baseline clustering of IPV and calculate the intraclass correlation coefficient (ICC).

Model II included individual-level factors. Our variable selection process was based on the prelimanary bivaraite analyses. Thus, we retained all individual-level varaibles that showed associated (p<0.05) in Model II.

Model III included contextual-level variables. We incorporated contextual-level variables that were statistically significant from the bivariate analyses. These variables allowed us to examine the broader community factors influencing IPV risk beyond individual factors. Model IV included all the explanatory variables that were statistically significant from the preliminary bivariate analyses. These changes can be find from lines 166 to 178 in pages 9 to 10 of the main manuscript.

The mixed-effects regression analysis yielded results that included both fixed and random effects. The fixed-effect analysis revealed the correlation between the explanatory variables and intimate partner violence among married women. The results were reported as an adjusted odds ratio (aOR) and their corresponding 95% confidence intervals (CI). The random effects findings underscore the variations in intimate partner violence among married women. All four models utilised the intra-cluster correlation coefficient (ICC) values to evaluate the variation. All analyses were weighted using the svyset command in Stata, which integrates sample weights, various phases of clustered sampling, and stratification to accommodate the intricate peculiarities of the DHS dataset. In our research, we utilised the svyset command in Stata to provide the survey design settings, including sample weights, clustering, and stratification. This methodology is essential for obtaining unbiased estimates and accurate standard errors in analysing complex survey data. The DHS dataset provides sample weights that reflect the selection probability at every stage of the sampling process. By employing these weights, we ensured that our estimations appropriately reflect the sample population, rectifying any disproportionate sampling of specific subgroups. This is essential in our research to accurately represent the realities of marginalised communities. The svyset program allows us to account for the clustered attributes of the sampling strategy. This suggests that individuals within the same cluster (e.g., geographic regions or communities) may demonstrate more similarity to each other than to those in different clusters. By specifying the clustering variable in our study, we adjusted the standard errors to account for intra-cluster correlation, yielding more reliable statistical inferences. The DHS employs a stratified sampling procedure to ensure representation across various demographic and geographic categories. Integrating the stratification variable into our svyset command maintained the sample structure and ensured the generalisability of our findings to the broader population.

o Provide a justification for why only certain socio-demographic variables

were considered, and discuss if any variables were excluded due to

collinearity or missing data.

**Response:** The study utilized fourteen socio-demographic variables based on their availability in the dataset and their relevance as identified in prior research on Intimate Partner Violence (IPV) [21–24]. These variables were selected to capture a broad range of factors that influence IPV, including individual characteristics (e.g., age, educational level, employment status), partner-related factors (e.g., controlling behaviors, alcohol consumption), societal influences (e.g., exposure to family planning media, justification of spousal abuse, interparental violence), and contextual factors (e.g., wealth index, residential location, geographical region). Certain socio-demographic variables that are known to influence IPV were not included in this study due to their unavailability in the dataset. For example, variables such as mental health status, history of childhood abuse, and detailed relationship dynamics (e.g., length of marriage or partnership) were not available in the DHS dataset.

o The study mentions the use of &quot;Empirical

Bayesian Kriging (EBK)&quot; for spatial interpolation. The rationale behind

selecting this technique over others should be explicitly discussed.

**Response: The Bayesian approach to spatial Kriging offers significant advantages over traditional interpolation methods, such as Inverse Distance Weighting (IDW) or frequentist Kriging, particularly in the context of complex health outcome data. Notably, empirical Bayesian Kriging (EBK) excels in mitigating variability and predicting values in regions that remain unsampled by exploiting data points from neighboring locales. This technique harnesses the principles of spatial autocorrelation to discern spatial trends within the data and to forecast the underlying dependency structures[38]. The method incorporates simulations and subsetting paradigms for parameter estimation, deriving the semivariogram from existing data points, thereby enabling the estimation of values at unobserved locations using a unified semivariogram. It is crucial to acknowledge that this approach presumes the semivariogram calculated for the interpolation zone accurately represents the true semivariogram [39].**

**In comparative assessments against alternative spatial interpolation techniques, Bayesian Kriging has demonstrated superior performance tailored to our specific research imperatives. EBK has exhibited enhanced accuracy through the integration of auxiliary variables together with spatial autocorrelation[40]. Furthermore, Bayesian Kriging addresses the tendency of Ordinary Kriging to produce overly optimistic prediction variances by conceptualizing model parameters as random variables, which facilitates more robust uncertainty quantification[41].**

**The implementation of Bayesian spatial Kriging has profoundly influenced our findings, establishing it as an invaluable tool for the exploration of patterns and risk factors associated with IPV. Numerous studies have successfully employed this methodology to identify high-risk areas in conjunction with pertinent neighborhood-level characteristics[42-45]. Moreover, this modeling approach adeptly identifies hotspots, regions characterized by uncertain estimates, and covariates that elucidate the spatial patterns of the outcome variable[43]. The Bayesian spatial modeling framework has enabled researchers to account for both fixed covariate effects and spatially structured random effects, thereby facilitating a comprehensive understanding of IPV patterns and informing targeted prevention strategies[44, 45]. We recognize that the application of Bayesian spatial techniques presents computational challenges and necessitates meticulous selection of prior distributions. To address these concerns, we undertook extensive sensitivity analyses to evaluate the influence of varying prior specifications on our study's findings.**

**3. Ethical Considerations:**

o The study states that &quot;Ethical clearance was not sought&quot; due to the

secondary nature of the data. However, IPV is a sensitive topic, and

there should be a discussion of how ethical considerations were

managed in handling potentially distressing content. o Explain whether

the data were anonymized and if informed consent was obtained in the

original survey.

**Response:** **Ethical clearance was not sought for this study as it utilized secondary data freely available in the public domain through the Demographic and Health Surveys (DHS) program. The DHS data are fully anonymized, with all personally identifiable information removed to protect the privacy of respondents. The original survey adhered to strict ethical protocols, including obtaining informed consent from all participants before data collection. Participants were made aware of the voluntary nature of their participation and were assured of the confidentiality of their responses. Additionally, the DHS program is reviewed and approved by the Institutional Review Board (IRB) of ICF International and, where applicable, by local IRBs in the respective countries where the surveys are conducted. While IPV is a sensitive topic, the DHS ensures that questions related to IPV are administered with care, typically in private settings to minimize distress or harm to respondents. More details about the ethical protocols followed in the DHS surveys can be accessed at [http://goo.gl/ny8T6X](http://goo.gl/ny8T6X).**

**4. Statistical Interpretation and Model Fit:**

o The reported odds ratios in Table 5 require a more nuanced

interpretation. Some variables (e.g., education, wealth index) were

found to be non-significant. The discussion should provide possible

explanations.

**Response:** **While some variables, such as partner controlling behavior, exposure to interparental violence, and husband's alcohol consumption, were found to have strong and statistically significant associations with IPV, others, including educational attainment and wealth index, were non-significant in the adjusted models. These non-significant findings warrant further exploration.**

**For educational attainment, the lack of statistical significance may reflect a complex relationship between education and IPV. While higher education levels are often associated with increased empowerment and awareness of rights, they may not necessarily translate into reduced IPV, particularly in contexts where cultural norms and societal attitudes toward gender roles are deeply entrenched. It is possible that education alone is insufficient to counteract structural inequalities or challenge patriarchal norms that perpetuate IPV. Additionally, the protective effect of education might be offset by other factors, such as economic dependency or limited access to resources, which could leave women vulnerable to IPV despite their educational attainment.**

**Similarly, the wealth index did not show significant associations with IPV in the adjusted models. This finding may be explained by the fact that IPV occurs across all socioeconomic strata, and wealth alone may not shield women from abuse. In some cases, wealthier households may experience IPV that is less visible or reported due to stigma or fear of social repercussions. Conversely, poorer households may face higher levels of stress and conflict, but these dynamics might already be captured by other variables in the model, such as partner controlling behavior or justification of wife-beating. The non-significant association could also indicate that wealth is not the primary determinant of IPV in the Sierra Leonean context, where other sociocultural factors may play a more dominant role.**

o Discuss the model’s goodness-of-fit (e.g., AIC, BIC, likelihood ratio

tests) and justify why the final model was chosen.

**Response:** **The goodness-of-fit of the models presented in Table 5 was assessed using several statistical metrics, including the Akaike Information Criterion (AIC), log-likelihood values, and Wald chi-square tests. These metrics provide insight into the relative quality of each model and its ability to explain the observed data.**

**1. Akaike Information Criterion (AIC):**

**The AIC values progressively decreased across the models, indicating improved model fit as additional variables were included. The empty model (Model I) had the highest AIC value [4672.93], reflecting its limited explanatory power due to the absence of covariates. Model IV, the final model, had the lowest AIC value [3990.38], suggesting that it provided the best fit to the data among the models. A lower AIC value indicates a better balance between model complexity and goodness-of-fit, as it penalizes unnecessary complexity.**

**2. Log-Likelihood:**

**Similarly, the log-likelihood values improved across the models, with Model IV having the highest log-likelihood [-1968.19]. Higher log-likelihood values indicate that the model is better at predicting the observed data. This improvement reflects the inclusion of additional covariates in Model IV, which enhanced its explanatory power.**

**3. Wald Chi-Square Tests:**

**The Wald chi-square test results demonstrate significant improvements in model fit as covariates were added. For instance, Model II had a Wald chi-square value of [317.55***], while Model IV showed the highest value of [364.31***], indicating that the inclusion of additional variables significantly contributed to explaining the variance in IPV.**

**4. Random Effects:**

**Intra-Class Correlation (ICC) values decreased slightly across the models, from 0.24 in Model I to 0.22 in Model IV. This reduction suggests that the inclusion of individual and contextual covariates in the final model accounted for some of the unexplained variance at the cluster level, improving the overall fit.**

**Justification for Choosing the Final Model (Model IV)**

**Model IV was chosen as the final model because it demonstrated the best goodness-of-fit metrics (lowest AIC, highest log-likelihood, and highest Wald chi-square value) while accounting for both individual-level and contextual factors associated with IPV. The inclusion of key covariates, such as partner controlling behavior, justification of wife-beating, exposure to interparental violence, and husband’s alcohol consumption, significantly enhanced the model’s explanatory power. These variables are well-supported in the literature as critical determinants of IPV, making Model IV the most comprehensive and theoretically sound choice.**

**While adding more covariates could further improve the model fit, the balance between parsimony and explanatory power was carefully considered. Model IV provides robust insights into the factors associated with IPV while avoiding overfitting or unnecessary complexity. Therefore, it was selected as the final model for interpretation and discussion.**

**5. Comparative Analysis with Existing Literature:**

o The introduction and discussion reference global and regional IPV

prevalence rates, but there is limited comparison with prior studies in

Sierra Leone. o The authors should engage more critically with existing

research on IPV determinants in Sub-Saharan Africa and discuss how

their findings align or differ from past studies.

**Response:** **Thank you for your insightful feedback. We appreciate your suggestion to engage more critically with existing research on IPV determinants in Sub-Saharan Africa and to provide greater comparisons with prior studies in Sierra Leone. We believe the current manuscript addresses these points comprehensively, as detailed below:**

**1. Comparison with Prior Studies in Sierra Leone:**

**The discussion section already includes comparisons with multiple studies conducted in Sierra Leone. For instance, we referenced a multicountry study that reported an IPV prevalence of 59.9% in Sierra Leone, which aligns closely with the findings of this study (56%). Additionally, we compared our results with a study on the prevalence, correlates, and trends of IPV in Sierra Leone, which reported a lifetime IPV prevalence of 60.8%, slightly exceeding the prevalence found in our study. These comparisons highlight consistency with prior research in Sierra Leone while situating our findings within the broader context of IPV prevalence in the country. Furthermore, we critically examined factors contributing to the high IPV prevalence in Sierra Leone, such as weak judiciary systems, entrenched cultural norms, socioeconomic challenges, and the impacts of health emergencies like Ebola and COVID-19, which align with findings from previous studies.**

**2. Engagement with Research on IPV Determinants in Sub-Saharan Africa:**

**The discussion section extensively compares our findings with studies conducted in other Sub-Saharan African countries. For example, we cited a multicountry analysis involving 26 Sub-Saharan African countries, noting similarities and differences in IPV prevalence rates and types of violence (e.g., emotional, physical, and sexual). Additionally, we referenced studies from Uganda and East African countries to contextualize our findings on IPV determinants, such as partner controlling behavior, justification of wife beating, and alcohol consumption. For instance, we discussed how cultural traditions, such as dowry practices in Uganda, may explain the association between controlling behavior and IPV, drawing parallels with Sierra Leone’s cultural practices. Similarly, we compared our findings on alcohol consumption and interparental violence with studies conducted in Sub-Saharan Africa, emphasizing the consistency of these risk factors across the region while highlighting the unique socio-cultural and historical context of Sierra Leone.**

**3. Alignment and Differences with Past Studies:**

**We critically engaged with existing research by highlighting both similarities and differences in IPV determinants. For instance, we noted that women aged 25–29 years in Sierra Leone had higher odds of experiencing IPV, which contrasts with findings from East African countries where this age group was not significantly associated with IPV. We attributed this difference to unique economic challenges and societal norms in Sierra Leone, such as stronger commitments to marriage and cohabitation, which may amplify IPV risk. Additionally, we discussed how cultural norms justifying wife beating and exposure to interparental violence are consistent risk factors across Sub-Saharan Africa, while emphasizing the exacerbating effects of Sierra Leone’s prolonged civil war and health emergencies on IPV prevalence.**

**By providing detailed comparisons with prior studies in Sierra Leone and critically engaging with regional research on IPV determinants, the manuscript situates its findings within a broader context and contributes valuable insights to the literature. We hope these clarifications address your concerns, and we remain open to further suggestions to strengthen the manuscript.**

**6. Implications and Recommendations:**

o The conclusion suggests policy interventions but remains somewhat generic. More concrete, actionable recommendations are needed. oConsider discussing how geographic mapping of IPV could be used for targeted interventions by local authorities and NGOs. o Address the feasibility of implementing policy changes in Sierra Leone’s socio-

political context.

**Response:** **Policy and Practice Implications**

**The findings of this study underscore the urgent need for targeted interventions to address intimate partner violence (IPV) in Sierra Leone, where prevalence remains alarmingly high at 56%. Geographic mapping of IPV prevalence provides a valuable opportunity for local authorities and NGOs to prioritize resources and implement interventions in hotspot districts such as the Western and Northwestern provinces. This tool can guide the allocation of funding, deployment of community-based education campaigns, and establishment of IPV support centers in high-risk areas. Meanwhile, cold spot districts, where IPV prevalence is comparatively lower, can serve as models for best practices in prevention and response strategies.**

**Strengthening the enforcement of existing IPV laws is critical to addressing the issue. While Sierra Leone has legal frameworks to combat violence against women, enforcement remains weak due to limited resources and societal acceptance of IPV. Policymakers should focus on building the capacity of law enforcement agencies through training on IPV sensitivity and the establishment of specialized units to handle IPV cases. Complementary public awareness campaigns can educate communities on the legal consequences of IPV, encourage survivors to seek justice, and reduce stigma.**

**Challenging cultural norms that justify wife-beating and controlling behaviors among partners is essential for long-term change. Community-based initiatives, such as dialogues with traditional leaders, religious figures, and local influencers, can help shift societal attitudes. Engaging men and boys through gender-transformative education programs can further reduce controlling behaviors and promote healthier relationships. These efforts should be complemented by programs aimed at breaking the cycle of intergenerational violence. Women exposed to interparental violence are at higher risk of experiencing IPV themselves, so trauma-informed care, counseling services, and parenting education are necessary to address this issue. Schools and community centers can also serve as safe spaces for children and adolescents to learn conflict resolution skills and reduce the likelihood of perpetuating IPV as adults.**

**Region-specific strategies are vital to addressing the disparities in IPV prevalence across Sierra Leone. In hotspot districts, interventions should include establishing IPV response centers equipped with legal, psychological, and medical support services. Mobile clinics can be deployed to provide services in remote areas. In contrast, cold spot districts can document and share their successful IPV prevention strategies for replication in other regions. Multi-sectoral collaboration is equally important, as IPV prevention must be integrated into broader health, education, and social programs. For example, maternal and child health services can screen for IPV and provide referrals to support services. A coordinated response involving government ministries, NGOs, and international organizations is essential for ensuring sustainability and impact.**

**Feasibility of Policy Implementation in Sierra Leone’s Socio-Political Context**

**Implementing these recommendations requires careful consideration of Sierra Leone’s socio-political realities, including limited resources, weak institutional capacity, and deeply entrenched cultural norms. To enhance feasibility, interventions should leverage existing community structures, such as the network of community health workers and local leaders, who can be trained to deliver IPV education and support services. Utilizing these structures reduces costs and ensures that interventions are culturally appropriate and accessible.**

**Strengthening political will is also essential. Advocacy efforts should focus on engaging policymakers and government officials to prioritize IPV prevention as a public health and human rights issue. Demonstrating the economic and social costs of IPV, such as its impact on productivity and healthcare expenses, can help galvanize political action. Securing sustainable funding through partnerships with international donors and development partners will be critical for bridging gaps in government capacity. NGOs can also play a key role in providing technical assistance and resources for IPV prevention programs.**

**Finally, community ownership of interventions must be prioritized to ensure success and sustainability. Programs designed with input from local communities are more likely to succeed in Sierra Leone’s decentralized governance system. Community-driven initiatives that address IPV will resonate more strongly with the population and foster long-term change.**

**Conclusion**

**This study highlights the high prevalence of intimate partner violence (IPV) among women of childbearing age in Sierra Leone, with significant regional disparities and associated risk factors. Geographic mapping of IPV prevalence provides a valuable tool for targeted interventions, enabling policymakers, local authorities, and NGOs to focus resources on hotspot districts such as the Western and Northwestern provinces. Concrete recommendations include strengthening enforcement of IPV laws, challenging cultural norms that justify violence, providing trauma-informed care to women exposed to interparental violence, and integrating IPV prevention into health and social services.**

**To ensure feasibility, interventions must leverage existing community structures, build political will, and secure sustainable funding through partnerships with international donors and NGOs. Community ownership of programs will be critical to addressing IPV in Sierra Leone’s socio-political context. Multi-sectoral collaboration and region-specific strategies are essential to reducing IPV and improving the safety and well-being of women across the country.**

**Minor Concerns and Suggestions:**

1. Grammar and Clarity:

o There are minor grammatical issues throughout the manuscript. A

thorough proofreading is recommended. o Example: In the results

section, the sentence &quot;The study revealed an overall IPV prevalence of

56%, with physical, sexual, and emotional violence accounting for

38%2%, 6.2%, and 45.9%, respectively.&quot; should be corrected for clarity.

**Response:** **The study revealed an overall IPV prevalence of 56%, with physical violence accounting for 38.2%, sexual violence for 6.2%, and emotional violence for 45.9%.**

**2. Figure and Table Presentation:**

o Some tables (e.g., Table 3) are dense and difficult to interpret. Consider

simplifying them by only including significant variables or restructuring

them for better readability. I suggest replacing Table 3 with a forest

plot, as it offers a more intuitive and visually informative

presentation of the data. This will enhance clarity and improve the

interpretability of the results. (sample forest plot attached)

**Response:** **Thank you for your suggestion regarding Table 3. We acknowledge that the table contains dense information; however, it was designed to provide a comprehensive overview of all variables analyzed in the study, including both significant and non-significant results. We believe this level of detail is essential for transparency and for allowing readers to assess the full scope of the findings.**

o Figures depicting spatial clusters of IPV are useful but should include a

more detailed legend and explanation of statistical significance.

**Response:** We appreciate the reviewer's valuable suggestion regarding improving the interpretability of our spatial analysis results. In response, we have enhanced all figures depicting IPV clusters.

**3. Terminology and Conceptual Consistency:**

o The manuscript sometimes alternates between &quot;IPV&quot; and &quot;domestic

violence&quot;—ensure consistency in terminology throughout.

**Response: We have now used IPV throughout the manuscript**

o The terms &quot;hotspots&quot; and &quot;cold spots&quot; should be clearly defined in the

methodology.

**Response:** **In this context, hotspots refer to districts with significantly high rates of IPV, where the prevalence is statistically higher than in surrounding areas. Conversely, cold spots refer to districts with significantly low rates of IPV, where the prevalence is statistically lower than in surrounding areas [36].**

**Conclusion:**

The manuscript makes a valuable contribution to the literature on IPV by

incorporating spatial analysis, but improvements in methodological clarity, statistical

interpretation, and policy recommendations are necessary. Addressing the above

concerns will strengthen the study’s impact and applicability. I encourage the authors

to revise the manuscript accordingly and provide detailed responses to the points

raised in their rebuttal letter.

**Response:** **Thank you for your valuable feedback on our manuscript. We appreciate your recognition of the contribution our study makes to the literature on intimate partner violence (IPV) through spatial analysis and acknowledge the importance of addressing the concerns raised. In response, we have enhanced the methodological clarity by defining key terms such as "hotspots" and "cold spots" and elaborating on the rationale for our spatial analysis techniques. We have also improved the statistical interpretation by providing a more detailed explanation of the significance of spatial autocorrelation measures and their implications for IPV clustering and dispersion. Additionally, we have strengthened the policy recommendations by offering actionable, context-specific strategies that address regional disparities in IPV prevalence and align with Sierra Leone’s socio-political framework. These revisions aim to improve the clarity, impact, and applicability of our study, and we look forward to your feedback on the revised manuscript.**
